# Abiraterone Acetate and Castration Resistant Ductal Adenocarcinoma of the Prostate

**DOI:** 10.1155/2014/508305

**Published:** 2014-05-07

**Authors:** Edgar Linden-Castro, Marcela Pelayo-Nieto, Alejandro Alias-Melgar, Daniel Espinosa-Perezgrovas, Ivan Ramirez-Galindo, Gabriel Catalan-Quinto

**Affiliations:** Urology Department, Centro Medico Nacional 20 de Noviembre, Félix Cuevas 540, Del Valle, Benito Juárez, 03229 Ciudad de México, DF, Mexico

## Abstract

Ductal adenocarcinoma of the prostate is a rare histological variant that only represents <1% of prostate tumors. This histological variant has several important clinical implications with respect to their evolution, clinical prognosis, and treatment. We report the case of a 64-year-old patient with ductal adenocarcinoma of the prostate, which progresses to castration-resistant prostate cancer, that was treated with abiraterone acetate with good clinical response, to our knowledge, the first case of ductal adenocarcinoma of the prostate in treatment with abiraterone acetate.

## 1. Introduction

Prostate cancer is a disease with a variable clinical course. Historically, Gleason score is an important factor in risk stratification. The role of the treatment and prognosis of nonacinar histological variants of prostate cancer is known from some variants, while not for others yet. Ductal adenocarcinoma of the prostate is a histological variant described in 1967, with some clinical implications that are not at all well established. Its prevalence in specimens obtained by transrectal prostate biopsy is 0.4%−1% and 5% for mixed ductal variants [1, 2]. There has been a considerable debate about whether the ductal adenocarcinoma of the prostate has a different behavior from its acinar counterpart. Most studies show that this histological variant reflects more aggressiveness and a worse prognosis, despite that the treatment is carried out in the same manner as of the acinar adenocarcinoma of the prostate.

## 2. Case Presentation

**Figure 1:**
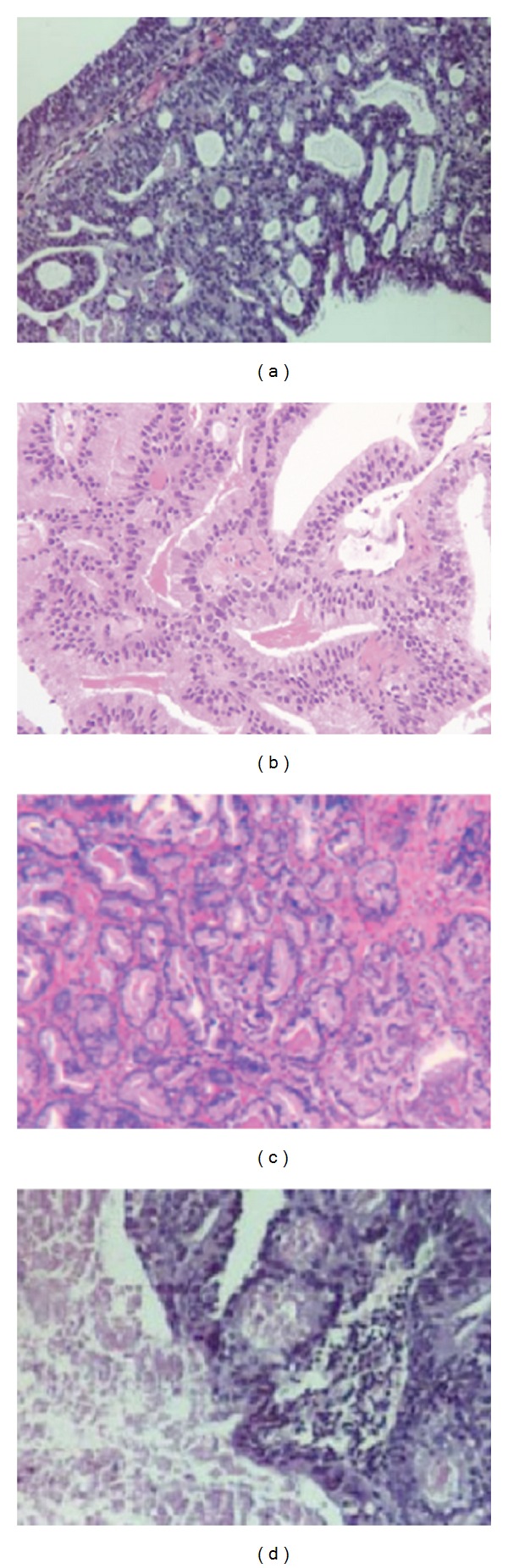
Examples of ductal adenocarcinoma of the prostate. (a) Stain of ductal prostate adenocarcinoma, (b) typical prostatic ductal adenocarcinoma, (c) acinar adenocarcinoma pattern, and (d) ductal adenocarcinoma of the prostate.

We present a clinical case report of a 64-year-old patient, with no history of chronic degenerative diseases. He began his current condition in 2007 with urinary voiding and storage symptoms, progressing to acute urinary retention. From that indication, we performed a TURP, obtaining prostate tissue with no evidence of malignancy. Eleven months after the surgical event, he developed dysuria and intermittent episodes of gross haematuria accompanied by elevated prostate-specific antigen levels (9.3 ng/mL); we performed a transrectal prostate biopsy, obtaining a histopathological result corresponding to prostate ductal adenocarcinoma (Figure 1).

We also performed another auxiliary diagnostic procedure, such as CT, with negative results to extraprostatic extension. For making the final histopathological diagnosis, IHC (immunohistochemistry) techniques were necessary, with the results being as follows: prostatic acid phosphatase (+), prostate-specific antigen (+), racemase (+), cytokeratin 7 (+), and cytokeratin 20 (−). Based on these results, the patient received a definitive management with three-dimensional conformal external radiation therapy (RT) plus hormone therapy (HT), with initial response for 24 months reaching a nadir value of 0.8 ng/mL.

**Figure 2:**
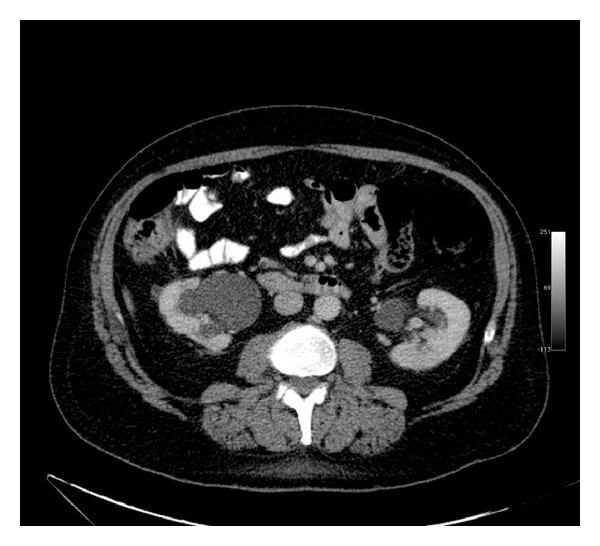
Abdominal computerized tomography revealing a right kidney dilatation.

After regular follow-up, right hydronephrosis secondary to obstructive uropathy was detected, requiring the placement of ipsilateral nephrostomy (Figure 2).

His condition evolved with a new rise on PSA level, now quantified in 7.8 ng/mL. An abdominopelvic CT revealed enlarged obturator lymph nodes in right hip (the greater of 1.5 × 1.2 cm). He also underwent a bone scan without detecting metastatic activity. From these findings, we decided to start androgen deprivation therapy, sustaining an initial biochemical response for 16 months. After that, there was another PSA elevation, the value being 18 ng/mL now, and another bone scan was performed, detecting metastatic activity.

Hormonal manipulation was performed twice, corroborating serum testosterone <0.069 ng/mL and persistent elevation of PSA, integrating the castration-resistance criteria according to the Prostate Cancer Clinical Trials Working Group 2 (PCWG2) criteria [3].

During the entire monitoring, adequate performance status of the patient is kept and his ECOG was 1, so we decided to start abiraterone acetate, 1000 mg every 24 hrs divided into 4 doses in combination with prednisone 5 mg every 12 hrs.

The initial decision involved monitoring the serum electrolytes at 14 and 28 days with values within the normal range. Likewise, there have been no documented adverse effects of clinical importance, only the presence of lower limb edema.

We observed that the patient had a PSA progression time of 9 months and radiographic progression-free survival of 11 months. The ECOG changed at 11 months to ECOG 2.

The time of initiation on cytotoxic chemotherapy was 14 months, after the start of abiraterone. We did not observe any shrinkage of the enlarged obturator lymph nodes after abiraterone initiation, but the patient is free of opioids. We compared our results with the COU-AA-302, and we found that our patient performed better than the prednisone-alone group.

## 3. Discussion

Most prostate tumors are acinar adenocarcinomas. The findings corresponding to other histological variants are quite infrequent and represent 5%–10% of prostate tumors. Pure ductal adenocarcinoma is a histological variant found in less than 1% of cases, while mixed ductal variants account for 5% of all cases [1, 2].

In 1967, Melicow et al. published the first case of ductal adenocarcinoma of the prostate describing the neoplasm's genesis from verumontanum with a histological appearance similar to endometrial adenocarcinoma [4, 5]. However, the aspects related to its origin have constituted a controversial theme since 1967.

Some clinically important aspects of ductal adenocarcinoma of the prostate include age of presentation at 60–70 years [6] and its association with increased urinary symptoms that differ from acinar adenocarcinoma, where it commonly presents obstructive symptoms or irritative ones, including macroscopic hematuria or acute urinary retention [4, 7, 8]. This way of presentation obeys the central location of the tumor. Unlike the majority of acinar adenocarcinoma patients, this variation tends to be asymptomatic and presents itself with elevated prostate-specific antigen [9].

Acinar adenocarcinoma of the prostate is commonly located in the peripheral zone (70%) with a minority arising from the transition zone (20%) or the central zone (1–5%), while ductal adenocarcinoma is identified in the central region due to the origin of the central prostatic ducts [9].

Morgan et al. demonstrated that men with ductal adenocarcinoma have 2.4 times more possibilities to have lower PSA of 4.0 ng/mL (OR 2.4, 95% CI 1.4–4.0, *P* = 0.001) [10]. The digital rectal examination is usually abnormal with an increase in size and hardness, without palpable nodules present; this is consistent with the depth and central region of the primary tumor [11, 12].

Bock and Bostwick hypothesized that prostate ductal adenocarcinoma arises from malignant degeneration of residual mullerian ducts, but ultrastructural studies have shown that the prostate ductal adenocarcinoma is a morphological variant of acinar prostate adenocarcinoma [13]. In this context, typically expressed prostatic immunohistochemical markers of prostate tissue include cytokeratin 7, PSA, PAP, AR, P63, all being positive, and less expression of AMACR and a low index of Ki67 [14].

The pathological appearance of ductal adenocarcinoma resembles an exophytic lesion or the presence of polypoid growth with whitish fronds in the urethra or nearby the verumontanum, as it is considered a remnant of mullerian ducts. From the clinical point of view, a prostatic urethra may be nodular or normal, and the tumor can be completely intraductal [11].

The diagnosis of ductal adenocarcinoma of the prostate is generally dependent on the histological characteristics with the appearance of a long stratified columnar epithelium and columnar structures located in the central region of the tumor [15]. This association supports the hypothesis that ductal adenocarcinoma of the prostate is not a single independent variant of prostate cancer, but it is a morphological variant of acinar carcinoma extending into the prostatic ducts. Bock and Bostwick questioned whether typical architectural characteristics of cribriform and papillary are sufficient to make this diagnosis, so they studied a series of specimens obtained by radical prostatectomy and found that the typical characteristics of this tumor were more frequent than expected. Therefore, they concluded that these characteristics were not unique to ductal carcinomas and suggested that the term should be used only for ductal carcinoma limited to ducts [13].

Ductal adenocarcinoma of the prostate is usually identified with Gleason pattern 4 [16]. In specimens obtained by radical prostatectomy, the pathological grade of ductal adenocarcinoma tends to be higher than in patients with acinar adenocarcinoma as well as the frequency of extraprostatic extension. Morgan et al. showed that these tumors tend to be of high grade, present themselves with more advanced disease (50% versus 32%, *P* < 0.001), and have poor differentiation and distant metastases (12% versus 3%, *P* < 0.001) compared to those with acinar carcinoma, leading to an increased mortality in these patients [10]. Ductal adenocarcinoma tends to metastasize to other distant sites such as lung, rectum, testis, and penis [8] thus men with ductal adenocarcinoma have a worse survival than those with acinar adenocarcinoma. Patients with localized ductal histology have 4 times the risk of PCa specific mortality compared to those with acinar histology (HR 3.9, 95% CI 2.6–5.8 disease), and even after multivariate analysis, the risk continues to be over 2-fold [10, 14]. Some studies show that a treatment for ductal adenocarcinoma that is maybe less sensitive than the standard treatment results in no difference to the treatment of acinar adenocarcinoma [16, 17].

This patient was treated in the context of a castration resistant prostate cancer with abiraterone acetate (1000 mg) and prednisone (5 mg twice daily). Abiraterone acetate was recently approved for the prechemotherapy treatment in patients with cancer castration resistant prostate. This is an inhibitor of the cytochrome P-450c17, an enzyme critical in the synthesis of extragonadal and testicular androgens. In the COU-AA-302, researchers found a median radiographic progression free survival of 16.5 months with abiraterone-prednisone and of 8.3 months with prednisone alone (*P* < 0.001). A median follow-up period of 22.2 months was also found along with the overall survival being improved with abiraterone-prednisone (HR, 0.75; 95% CI, 0.61 to 0.93; *P* = 0.01). However, the results did not cross the efficacy boundary. After we compared our results with these, we found that, even in castration resistant ductal adenocarcinoma of the prostate, which is a different histology of the cases reported in the COU-AA-302, treatment consisting of abiraterone-prednisone showed superiority over prednisone alone. This was with respect to time of the initiation of cytotoxic chemotherapy, opiate use for cancer-related pain, prostate-specific antigen progression, and decline in performance status [18]. However, it must take attention of early bone metastases or some other visceral structures and the potential use of other markers such as carcinoembryonic antigen [19].
